# Antioxidant activity, total phenolic and total flavonoid contents of whole plant extracts *Torilis leptophylla* L

**DOI:** 10.1186/1472-6882-12-221

**Published:** 2012-11-16

**Authors:** Naima Saeed, Muhammad R Khan, Maria Shabbir

**Affiliations:** 1 Department of Biochemistry, Faculty of Biological Sciences, Quaid-i-Azam University, Islamabad, 45320, Pakistan

**Keywords:** *Torilis leptophylla*, DPPH, Antioxidant, Oxidative stress, TBARS

## Abstract

**Background:**

The aim of this study was to screen various solvent extracts of whole plant of *Torilis leptophylla* to display potent antioxidant activity *in vitro* and *in vivo*, total phenolic and flavonoid contents in order to find possible sources for future novel antioxidants in food and pharmaceutical formulations.

**Material and methods:**

A detailed study was performed on the antioxidant activity of the methanol extract of whole plant of *Torilis leptophylla* (TLM) and its derived fractions {*n*-hexane (TLH), chloroform (TLC) ethyl acetate (TLE) *n*-butanol (TLB) and residual aqueous fraction (TLA)} by *in vitro* chemical analyses and carbon tetrachloride (CCl_4_) induced hepatic injuries (lipid peroxidation and glutathione contents) in male Sprague-Dawley rat. The total yield, total phenolic (TPC) and total flavonoid contents (TFC) of all the fractions were also determined. TLM was also subjected to preliminary phytochemical screening test for various constituents.

**Results:**

The total phenolic contents (TPC) (121.9±3.1 mg GAE/g extract) of TLM while total flavonoid contents (TFC) of TLE (60.9 ±2.2 mg RTE/g extract) were found significantly higher as compared to other solvent fractions. Phytochemical screening of TLM revealed the presence of alkaloids, anthraquinones, cardiac glycosides, coumarins, flavonoids, saponins, phlobatannins, tannins and terpenoids. The EC_50_ values based on the DPPH (41.0±1 μg/ml), ABTS (10.0±0.9 μg/ml) and phosphomolybdate (10.7±2 μg/ml) for TLB, hydroxyl radicals (8.0±1 μg/ml) for TLC, superoxide radicals (57.0±0.3 μg/ml) for TLM and hydrogen peroxide radicals (68.0±2 μg/ml) for TLE were generally lower showing potential antioxidant properties. A significant but marginal positive correlation was found between TPC and EC_50_ values for DPPH, hydroxyl, phosphomolybdate and ABTS, whereas another weak and positive correlation was determined between TFC and EC_50_ values for superoxide anion and hydroxyl radicals. Results of *in vivo* experiment revealed that administration of CCl_4_ caused a significant increase in lipid peroxidation (TBARS) while decrease in GSH contents of liver. In contrast, TLM (200 mg/kg bw) and silymarin (50 mg/kg bw) co-treatment effectively prevented these alterations and maintained the antioxidant status.

**Conclusion:**

Data from present results revealed that *Torilis leptophylla* act as an antioxidant agent due to its free radical scavenging and cytoprotective activity.

## Background

Since very old times, herbal medications have been used for relief of symptoms of disease [1]. Despite the great advances observed in modern medicine in recent decades, plants still make an important contribution to health care. Much interest, in medicinal plants however, emanates from their long use in folk medicines as well as their prophylactic properties, especially in developing countries. Large number of medicinal plants has been investigated for their antioxidant properties. Natural antioxidants either in the form of raw extracts or their chemical constituents are very effective to prevent the destructive processes caused by oxidative stress [2]. Although the toxicity profile of most medicinal plants have not been thoroughly evaluated, it is generally accepted that medicines derived from plant products are safer than their synthetic counterparts [3,4].

Substantial evidence has accumulated and indicated key roles for reactive oxygen species (ROS) and other oxidants in causing numerous disorders and diseases. The evidence has brought the attention of scientists to an appreciation of antioxidants for prevention and treatment of diseases, and maintenance of human health [5]. Human body has an inherent antioxidative mechanism and many of the biological functions such as the anti-mutagenic, anti-carcinogenic, and anti-aging responses originate from this property [6,7]. Antioxidants stabilize or deactivate free radicals, often before they attack targets in biological cells [8]. Recently interest in naturally occurring antioxidants has considerably increased for use in food, cosmetic and pharmaceutical products, because they possess multifacetedness in their multitude and magnitude of activity and provide enormous scope in correcting imbalance [9,10].

The role of free radical reactions in disease pathology is well established and is known to be involved in many acute and chronic disorders in human beings, such as diabetes, atherosclerosis, aging, immunosuppression and neurodegeneration [11]. An imbalance between ROS and the inherent antioxidant capacity of the body, directed the use of dietary and /or medicinal supplements particularly during the disease attack. Studies on herbal plants, vegetables, and fruits have indicated the presence of antioxidants such as phenolics, flavonoids, tannins, and proanthocyanidins. The antioxidant contents of medicinal plants may contribute to the protection they offer from disease. The ingestion of natural antioxidants has been inversely associated with morbidity and mortality from degenerative disorders [6]. Liver diseases remain a serious health problem. It is well known that free radicals cause cell damage through mechanisms of covalent binding and lipid peroxidation with subsequent tissue injury. Antioxidant agents of natural origin have attracted special interest because of their free radical scavenging abilities [12]. The use of medicinal plants with high level of antioxidant constituents has been proposed as an effective therapeutic approach for hepatic damages [13].

*Torilis leptophylla* belonging to the Apiaceae family [14] or more commonly known as Bristle fruit Hedge parsley, is a forb of the genus *Torilis*, distributed in Asia, Europe and North Africa. It is represented in Iran by nine species [15]. *T*. *leptophylla* has been used in folk medicine for the treatment of gastrointestinal illnesses in Iran and Pakistan. The plant is highly effective against some pathogens thus confirming its use as disinfectant or antiseptic [16].

The search for novel natural antioxidants of plant origin has ever since increased. It is not known which constituents of plant are associated in reducing the risk of chronic diseases, but antioxidants appear to play a major role in the protective effect of plant medicine. The present study was designed to investigate the TPC and TFC and to evaluate the antioxidant activities (*in vitro* and *in vivo*) of the various fractions of methanol extract of whole plant of *T*. *leptophylla*. The 95% methanol extract was also subjected to phytochemical screening to determine the presence of alkaloids, anthraquinones, cardiac glycosides, coumarins, flavonoids, saponins, phlobatannins, tannins and terpenoids.

## Methods

### Plant collection

The plant was collected in April 2011 from Islamabad, Pakistan. The plant material was botanically identified by Dr. Mir Ajab Khan, Department of Plant Sciences, Quaid-i-Azam University Islamabad. A voucher specimen (accession #023541) was deposited at the Herbarium of Pakistan Museum of Natural History Islamabad.

### Extract preparation

The fresh, whole plant (3 kg) was collected and shade dried to obtain 500 g dry sample which was later coarsely powdered in a Willy Mill to 60-mesh size and used for solvent extraction. For sample preparation, 500 g of dried sample were extracted twice (2000 ml for each) with 95% methanol at 25°C for 48 h and concentrated using a rotary evaporator (Panchun Scientific Co., Kaohsiung, Taiwan) under reduced pressure at 40°C to yield the TLM (11.5%). The residue was suspended in water (50 ml) and partitioned successively with *n*-hexane, chloroform, ethyl acetate, *n*-butanol (a total of two aliquots of 100 ml each) and soluble residual aqueous fraction yielding respectively the TLH (5.4%), TLC (4.3%), TLE (6.1%), TLB (4.8%) and TLA (8.2%) [17].

### Chemicals

Ascorbic acid; aluminum chloride, 2, 2^′^ - azino-bis-(3- ethylbenzothiazoline-6-sulphonic acid) (ABTS); ferric chloride (FeCl_3_); Folin-Ciocalteu; bovine serum albumin (BSA); potassium persulphate; 2,2^′^-diphenyl-1-picrylhydrazyl (DPPH); nitro blue tetrazolium (NBT); phenazine methosulphate (PMS); reduced glutathione (GSH); 1,2-dithio-bis nitro benzoic acid (DTNB); sulphosalicylic acid; thiobarbituric acid (TBA) and trichloroacetic acid (TCA) were purchased from Sigma Co. (St. Louis, MO, USA). Sulphuric acid; 2-deoxyribose; riboflavin; sodium carbonate (Na_2_CO_3_); sodium hydroxide (NaOH); sodium nitrite (NaNO_2_); disodium hydrogen phosphate (Na_2_HPO_4_) and hydrogen peroxide (H_2_O_2_) were obtained from Wako Co. (Osaka, Japan). Potassium ferricyanide [K_3_Fe (CN)_6_]; triflouroacetic acid; sodium dihydrogen phosphate (NaH_2_PO_4_) and all solvents *n*-hexane (99.8%); chloroform (99.8%); ethyl acetate (99.8%) and *n*-butanol (99.8%) used were of analytical grade and purchased from Merck Co. (Darmstadt, Germany). Distilled deionized water (dd. H_2_O) was prepared by Ultrapure TM water purification system (Lotun Co., Ltd., Taipei, Taiwan).

### *In vitro* studies

#### Antioxidant assays

Each sample was dissolved in 95% methanol to make a concentration of 1 mg/ml and then diluted to prepare the series concentrations for antioxidant assays. Reference chemicals were used for comparison in all assays.

### DPPH radical scavenging activity assay

The free radical scavenging activity of the fractions was measured *in vitro* by 2,2^′^- diphenyl-1-picrylhydrazyl (DPPH) assay according to the method described earlier [18,19]. The stock solution was prepared by dissolving 24 mg DPPH with 100 ml methanol and stored at 20°C until required. The working solution was obtained by diluting DPPH solution with methanol to attain an absorbance of about 0.98±0.02 at 517 nm using the spectrophotometer. A 3 ml aliquot of this solution was mixed with 100 μl of the sample at various concentrations (10 - 500 μg/ml). The reaction mixture was shaken well and incubated in the dark for 15 min at room temperature. Then the absorbance was taken at 517 nm. The control was prepared as above without any sample. The scavenging activity was estimated based on the percentage of DPPH radical scavenged as the following equation:

(1) $$\text{Scavenging}\;\text{effect}\;\left(\%\right)=\left[\left(\text{control}\;\text{absorbance}-\text{sample}\;\text{absorbance}\right)/\left(\text{control}\;\text{asbsorbance}\right)\right]\times \;100$$

### Superoxide anion scavenging assay

The assay for superoxide anion radical scavenging activity was supported by riboflavin-light-NBT system [20]. Briefly, 1 ml of sample was taken at different concentrations (25 to 500 μg/ml) and mixed with 0.5 ml of phosphate buffer (50 mM, pH 7.6), 0.3 ml riboflavin (50 mM), 0.25 ml PMS (20 mM), and 0.1 ml NBT (0.5 mM). Reaction was started by illuminating the reaction mixture using a fluorescent lamp. After 20 min of incubation, the absorbance was measured at 560 nm. Ascorbic acid was used as standard. The scavenging ability of the plant extract was determined by the following equation:

(2) $$\text{Scavenging}\;\text{effect}\;\left(\%\right)=\left(1-\text{absorbance}\;\text{of}\;\text{sample}/\text{absorbance}\;\text{of}\;\text{control}\right)\times \;100$$

### Phosphomolybdate assay (total antioxidant capacity)

The total antioxidant capacity of the fractions was determined by phosphomolybdate method using ascorbic acid as a standard [21]. An aliquot of 0.1 ml of sample solution was mixed with 1 ml of reagent solution (0.6 M sulphuric acid, 28 mM sodium phosphate and 4 mM ammonium molybdate). The tubes were capped and incubated in a water bath at 95°C for 90 min. After the samples had cooled to room temperature, the absorbance of the mixture was measured at 765 nm against a blank. A typical blank contained 1 ml of the reagent solution and the appropriate volume of the solvent and incubated under the same conditions. Ascorbic acid was used as standard. The antioxidant capacity was estimated using following formula:

(3) $$\text{Antioxidant}\;\text{effect}\;\left(\%\right)=\left[\left(\text{control}\;\text{absorbance}-\text{sample}\;\text{absorbance}\right)/\left(\text{control}\;\text{absorbance}\right)\right]\times \;100$$

### Hydroxyl radical scavenging assay

Hydroxyl radical scavenging activity was measured by the ability of the different fractions of *T*. *leptophylla* extract to scavenge the hydroxyl radicals generated by the Fe^3+^-ascorbate-EDTA-H_2_O_2_ system (Fenton reaction) [22]. The reaction mixture contained; 500 μl of 2-deoxyribose (2.8 mM) in phosphate buffer (50 mM, pH 7.4), 200 μl of premixed ferric chloride (100 mM) and EDTA (100 mM) solution (1:1; v/v), 100 μl of H_2_O_2_ (200 mM) with or without the extract solution (100 μl). The reaction was triggered by adding 100 μl of 300 mM ascorbate and incubated for 1 h at 37°C. 0.5 ml of the reaction mixture was added to 1 ml of TCA (2.8%; w/v; aqueous solution), then 1 ml of 1% aqueous TBA were added to the reaction mixture. The mixture was heated for 15 min on a boiling water bath. After the mixture being cooled the absorbance at 532 nm was noted against a blank (the same solution but without reagent). The scavenging activity on hydroxyl radical was calculated as follows:

(4) $$\text{Scavenging}\;\text{activity}\left(\%\right)=\left(1-\text{absorbance}\;\text{of}\;\text{sample}/\text{absorbance}\;\text{of}\;\text{control}\right)\times \;100$$

### Hydrogen peroxide scavenging activity

Hydrogen peroxide solution (2 mM) was prepared in 50 mM phosphate buffer (pH 7.4). Aliquots (0.1 ml) of different fractions was transferred into the test tubes and their volumes were made up to 0.4 ml with 50 mM phosphate buffer (pH 7.4) After addition of 0.6 ml hydrogen peroxide solution, tubes were vortexed and absorbance of the hydrogen peroxide at 230 nm was determined after 10 min, against a blank [23]. The abilities to scavenge the hydrogen peroxide were calculated using the following equation:

(5) $$\text{Hydrogen}\;\text{peroxide}\;\text{scavenging}\;\text{activity}=\left(1-\text{absorbance}\;\text{of}\;\text{sample}/\text{absorbance}\;\text{of}\;\text{sample}\right)\times \;100$$

### ABTS radical scavenging activity

The 2,2^′^-azinobis (3-ethylbenzthiazoline-6-sulphonic acid), commonly called ABTS cation scavenging activity was performed [24]. Briefly, ABTS solution (7 mM) was reacted with potassium persulfate (2.45 mM) solution and kept for overnight in the dark to yield a dark coloured solution containing ABTS radical cations. Prior to use in the assay, the ABTS radical cation was diluted with 50% methanol for an initial absorbance of about 0.70±0.02 at 745 nm, with temperature control set at 30°C. Free radical scavenging activity was assessed by mixing 300 μl of test sample with 3.0 ml of ABTS working standard in a microcuvette. The decrease in absorbance was measured exactly one minute after mixing the solution, then up to 6 min. The percentage inhibition was calculated according to the formula:

(6) $$\text{Scavenging}\;\text{effect}\;\left(\%\right)=\left[\left(\text{control}\;\text{absorbance}-\text{sample}\;\text{absorbance}\right)/\left(\text{control}\;\text{asbsorbance}\right)\right]\times \;100$$

The antioxidant capacity of test samples was expressed as EC_50_ (anti-radical activity)_,_ the concentration necessary for 50% reduction of ABTS [25].

### Reducing power

The reducing power was based on Fe (III) to Fe (II) transformation in the presence of the solvent fractions [26]. The Fe (II) can be monitored by measuring the formation of Perl’s Prussian blue at 700 nm. Various concentrations of the sample (2 ml) were mixed with 2 ml of phosphate buffer (0.2 M, pH 6.6) and 2 ml of potassium ferricyanide (10 mg/ml). The mixture was incubated at 50°C for 20 min followed by addition of 2 ml of trichloroacetic acid (100 mg/l). The mixture was centrifuged at 3000 rpm for 10 min to collect the upper layer of the solution. A volume of 2 ml from each of the mixture earlier mentioned was mixed with 2 ml of distilled water and 0.4 ml of 0.1% (w/v) fresh ferric chloride. After 10 min reaction, the absorbance was measured at 700 nm. Higher absorbance of the reaction mixture indicates a higher reducing power.

### Estimation of total phenolic content

The total phenolic content was determined by the spectrophotometric method [27]. In brief, a 1 ml of sample (1 mg/ml) was mixed with 1 ml of Folin-Ciocalteu’s phenol reagent. After 5 min, 10 ml of a 7% Na_2_CO_3_ solution was added to the mixture followed by the addition of 13 ml of deionized distilled water and mixed thoroughly. The mixture was kept in the dark for 90 min at 23°C, after which the absorbance was read at 750 nm. The TPC was determined from extrapolation of calibration curve which was made by preparing gallic acid solution. The estimation of the phenolic compounds was carried out in triplicate. The TPC was expressed as milligrams of gallic acid equivalents (GAE) per g of dried sample.

### Estimation of total flavonoid content

Total flavonoid content was determined following a method by Park *et al* (2008) [28]. In a 10 ml test tube, 0.3 ml of extracts, 3.4 ml of 30% methanol, 0.15 ml of NaNO_2_ (0.5 M) and 0.15 ml of AlCl_3_.6H_2_O (0.3 M) were mixed. After 5 min, 1 ml of NaOH (1 M) was added. The solution was mixed well and the absorbance was measured against the reagent blank at 506 nm. The standard curve for total flavonoids was made using rutin standard solution (0 to 100 mg/l) under the same procedure as earlier described. The total flavonoids were expressed as milligrams of rutin equivalents per g of dried fraction.

### Phytochemical screening of TLM

Phytochemical screening of TLM for the presence of alkaloids, anthraquinones, cardiac glycosides, coumarins, flavonoids, saponins, phlobatannins, tannins and terpenoids was carried out.

### Test for alkaloids

0.4 g of TLM was stirred with 8 ml of 1% HCl and the mixture was warmed and filtered [29]. 2 ml of filtrate was treated separately with (a) with few drops of potassium mercuric iodide (Mayer’s reagent) and (b) potassium bismuth (Dragendroff’s reagent). Turbidity or precipitation with either of these reagents was taken as evidence for existence of alkaloids.

### Test for saponins

The ability of saponins to produce emulsion with oil was used for the screening test [29]. 20 mg of TLM was boiled in 20 ml of distilled water in a water bath for five min and filtered. 10 ml of the filtrate was mixed with 5 ml of distilled water and shaken vigorously for froth formation. 3 drops of olive oil were mixed with froth, shaken vigorously and observed for emulsion development.

### Test for terpenoids

Presence of terpenoids in TLM was carried out by taking 5 ml (1 mg/ml) of TLM and mixed with 2 ml of chloroform, followed by 3 ml of concentrated H_2_SO_4_. A reddish brown colouration of the interface confirmed the presence of terpenoids [29].

### Test for anthraquinones

200 mg of TLM was boiled with 6 ml of 1% HCl and filtered. The filtrate was shaken with 5 ml of benzene, filtered and 2 ml of 10% ammonia solution was added to the filtrate. The mixture was shaken and the presence of a pink, violet or red colour in the ammoniacal phase indicated the presence of free hydroxyl anthraquinones [30].

### Cardiac glycosides determination

5 ml (10 mg/ml in methanol) of TLM was mixed with 2 ml of glacial acetic acid having one drop of FeCl_3_ solution. To the mixture obtained 1 ml of concentrated H_2_SO_4_ was added to form a layer. The presence of brown ring of the interface indicated deoxy sugar characteristic of cardiac glycosides [30].

### Test for coumarins

In a small test tube, 300 mg of TLM was covered with filter paper moistened with 1 N NaOH. The test tube was placed for few minutes in a boiling water bath. After removing the filter paper it was examined under UV light, yellow florescence indicated the presence of coumarins [30].

### Test for phlobatannins

80 mg of TLM was boiled in 1% aqueous hydrochloric acid; the deposition of a red precipitate indicated the presence of phlobatannins [30].

### Test for flavonoids

50 mg of TLM was suspended in 100 ml of distilled water to get the filtrate. 5 ml of dilute ammonia solution was added to 10 ml of filtrate followed by few drops of concentrated H_2_SO_4_. Presence of flavonoids was confirmed by yellow colouration [31].

### Test for tannins

50 mg of TLM was boiled in 20 ml of distilled water and filtered. A few drops of 0.1% FeCl_3_ was added in filtrate and observed for colour change; brownish green or a blue-black colouration was taken as evidence for the presence of tannins [31].

### *In vivo* studies

#### Animals and treatment

Studies were carried out using male Sprague Dawley rats weighing 180±10 g. The animals were grouped and housed in polyacrylic cages with not more than six animals per cage and maintained under standard laboratory conditions. They had free access to standard diet and fresh water *ad libitum*. For acute toxicity studies, 30 rats were divided randomly into 5 groups, each comprising 6 animals. Group (I) the controls received only vehicles; olive oil (0.5 ml/kg bw) and DMSO (0.5 ml/kg bw) and fed with a normal diet for 7 days. Group II (induction controls) received single dose of CCl_4_ (CCl_4_ + Olive oil in 1:1 ratio; 2 ml/kg bw; i.p) on day 1 and day 7 of the experiment. Group ІІІ and IV received TLM (200 mg/kg bw; i.p) and silymarin (25 mg/kg, i.p) as the standard reference drug, once in a day for 7 days respectively along with the intraperitoneal administration of CCl_4_ on day 1 and 7. Group V received only TLM (200 mg/kg; ip) once in a day for 7 days. At the end of 7 days, 24 h of the last treatment, all the animals were anesthetized in an ether chamber. The liver was removed and placed at 4°C after perfusion with ice cold saline. Intraperitoneal route of administration is selected because it is more rapid and predictable absorption than oral administration. This route is also advantageous because the drug is not inactivated or destroyed as may happen in the gastrointestinal tract and usually smaller doses are required because large concentrations of drug is achieved at the affected site. All experimental procedures involving animals were conducted in accordance with the guidelines of National Institutes of Health (NIH guidelines Islamabad, Pakistan). The study protocol was approved (No. 0241) by Ethical Committee of Quaid-i-Azam University, Islamabad.

### Determination of *in vivo* antioxidant activity

10% homogenate of liver tissue was prepared in 100 mM KH_2_PO_4_ buffer containing 1 mM EDTA (pH 7.4) and centrifuged at 12,000 × g for 30 min at 4°C. The supernatant was collected and used for the following experiments as described below.

### Determination of thiobarbituric acid reactive substances (TBARS)

Malondialdehyde in liver homogenate was determined by reaction with thiobarbituric acid (TBA). Briefly; 1.0 ml reaction assay was consisted of 0.58 ml phosphate buffer (0.1 M; pH 7.4), 0.2 ml liver supernatant and following addition of 1.0 ml 0.67% thiobarbituric acid, all the tubes were placed in boiling water bath for 20 min and then shifted to crushed ice-bath before centrifuging at 2500 × g for 10 min. The amount of TBARS formed in each of the samples was assessed by measuring optical density of the supernatant at 535 nm using spectrophotometer against a reagent blank. The results were expressed as nM TBARS/min/mg tissue at 37°C using molar extinction coefficient of 1.56 ×10^5^ M ˉ^1^cmˉ^1^[32].

### Determination of reduced glutathione (GSH)

Reduced glutathione in liver homogenate was determined by reaction with 1,2-dithio-bis nitro benzoic acid (DTNB). Briefly, 1.0 ml of supernatant was precipitated with 1.0 ml of (4%) sulphosalicylic acid. The samples were kept at 4°C for 1 h and then centrifuged at 1200 × g for 20 min at 4°C. The total volume of 3.0 ml assay mixture contained 0.1 ml filtered aliquot, 2.7 ml phosphate buffer (0.1 M; pH 7.4) and 0.2 ml of 1,2-dithio-bis nitro benzoic acid (DTNB, 100 mM). The yellow colour developed was read immediately at 412 nm on a Smart- SpecTM plus Spectrophotometer. It was expressed as μM GSH/g tissue [33].

### Statistical analysis

Data are expressed as mean ± SD from three separate observations. For in vitro antioxidant assays one way ANOVA test followed by Tukey’s test (P < 0.05) was used to analyze the differences among EC_50_ of various fractions for different antioxidant assays. The EC_50_ values were determined using the Graph Pad Prism 5 software. Data on biochemical investigations of *in vivo* experiments were analyzed by one-way (ANOVA) and the group means were compared by Dunnet’s Multiple Range Test. A probability of P < 0.05 was considered as significant.

## Results

### Phytochemical screening

Phytochemical screening of TLM demonstrated the presence of alkaloids, anthraquinones, cardiac glycosides, coumarins, flavonoids, saponins, phlobatannins, tannins and terpenoids.

### In vitro antioxidant activity

Antioxidant capacity of TLM and its derived fractions was examined using seven different assays.

### DPPH radical scavenging activity

**Figure 1:**
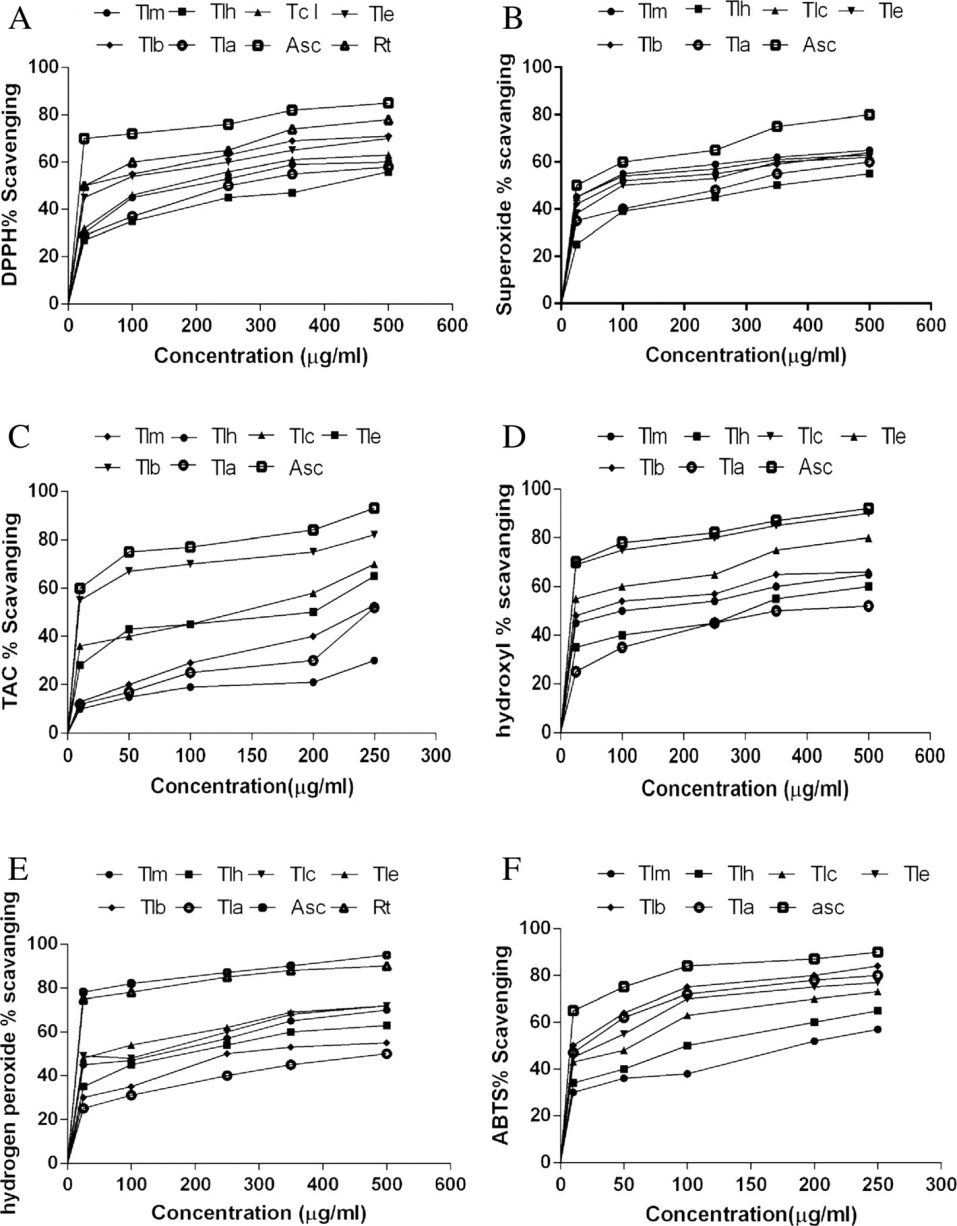
**Antioxidant activities of different extracts from the methanol extract of whole plant of *T. leptophylla *by different solvents at various concentrations.** Each value represents a mean ± SD (n = 3): (**A**) DPPH radical scavenging activity, (**B**) Superoxide radical scavenging activity, (**C**) Total antioxidant capacity, (**D**) Hydroxyl radical scavenging activity, (**E**) Hydrogen peroxide radical scavenging activity, (F) ABTS radical scavenging activity. Tlm., methanol fraction, Tlh., *n*-hexane fraction, Tlc., chloroform fraction, Tle., ethyl acetate fraction, Tlb., *n*-butanol fraction, Tla., residual aqueous fraction, Asc., ascorbic acid, Rt., rutin.

Figure 1A shows that the scavenging effects of samples on DPPH radical and were in the following order: TLB > TLE > TLC > TLM >TLA > TLH. The EC_50_ values of scavenging DPPH radicals for the TLB and TLE were 41.0±1 and 62.0±2 μg/ml, respectively (Table 1). Though the antioxidant potential of fractions was found to be low (P < 0.05) than those of ascorbic acid and rutin, the study revealed that TLB and TLE have prominent antioxidant activity; the presence of phenolic compounds (containing phenolic hydroxyls) are mainly found in these two fractions and could be attributable to the observed high antiradical properties of these fractions.

**Table 1:** **Radical scavenging activities of *T. leptophylla *fractions at different concentrations**

**EC**_**50**_**values(μg/ml) of radical scavenging**						
**Plant extracts/chemical**	**DPPH radical**	**Superoxide radical**	**Phosphomolybdate Assay**	**Hydroxyl radical**	**Hydrogen peroxide**	**ABTS radical**
Methanol extract	189±4^e^	55.0±0.3^d^	236±1^e^	76.0±2^d^	130±5^e^	179±3^e^
*n*-Hexane fraction	395±5^f^	370±4^f^	247±3^e^	281±2^e^	200±2^e^	50.0±1^c^
Chloroform fraction	162±3^e^	68.0±2^d^	135±4^e^	8.0±1^a^	122±5^e^	57.0±2^d^
Ethyl acetate fraction	62.0±2^d^	145±1^e^	196±4^e^	27.0±1^b^	68.0±2^d^	34.0±3^b^
*n*-Butanol fraction	41.0±1^c^	92.0±1^d^	10.7±2^a^	62.0±2^d^	254±3^e^	10.0±0.9^a^
Residual aqueous fraction	264±4^e^	269±3^e^	237±3^e^	345±4^f^	431±5^f^	13.0±0.2^a^
Ascorbic acid	10.0±3^a^	34.0±3^b^	8.0±0.7^a^	6.0±2^a^	9.0±0.3^a^	8.0±1^a^
Rutin	29.0±1^b^	-	-	-	10.0±0.3^a^	-

Each value in the table is represented as mean ± SD (n = 3). Values in the same column followed by a different letter (^a-f^) are significantly different (p< 0.05). -, not determined.

### Superoxide radical scavenging activity

Superoxide radical is considered a major biological source of reactive oxygen species [34]. Although superoxide anion is a weak oxidant, it gives rise to generation of powerful and dangerous hydroxyl radicals as well as singlet oxygen, both of which contribute to oxidative stress [35]. The superoxide radical scavenging effect of different fractions was compared with the same doses of ascorbic acid ranging from 25 - 500 μg/ml. The EC_50_ values in superoxide scavenging activities were in the order of TLM > TLC > TLB > TLE > TLA > TLH (Table 1). When compared to ascorbic acid; the superoxide scavenging activity of the extract was found to be low (P < 0.05). In spite of this TLM and TLC (EC_50_ 55.0±0.3 and 68.0±2 μg/ml respectively) behave as powerful superoxide anion scavengers that may include therapeutic use against oxidative stress.

### Phosphomolybdate assay

The phosphomolybdate method is quantitative, since the total antioxidant capacity (TAC) is expressed as ascorbic acid equivalents. The antioxidant capacity of various solvent fractions of *T*. *leptophylla* was found to decrease in this order: TLB > TLC > TLE > TLM > TLA > TLH fraction (Table 1; Figure 1C). All results showed antioxidant activity in dose dependent manner at concentration 25 to 250 μg/ml. The EC_50_ value of antioxidant capacity for the TLB (10.7±2 μg/ml) was most pronounced (P < 0.05) than TLC (135±4 μg/ml) and TLE (196±4 μg/ml) (Table 1). Strong antioxidant activity of TLB statistically similar to ascorbic acid indicates strong antioxidants in this fraction and these could be attributable to the presence of phenolic compounds.

### Hydroxyl radical scavenging activity

Hydroxyl radical scavenging activity was quantified by measuring the inhibition of the degradation of 2-deoxyribose by the free radicals generated by the Fenton reaction. The hydroxyl radical scavenging activity of *T*. *leptophylla* extract and its derived fractions can be ranked as TLC > TLE > TLB > TLM > TLH and TLA (Table 1). All fractions showed antioxidant activity in dose dependent manner at concentration 25 - 500 μg/ml (Figure 1D). In the present investigation, the EC_50_ value of hydroxyl radical scavenging activity for the TLC and TLE was 8.0±1 and 27.0±1 μg/ml while for TLB was 62.0±2 μg/ml (Table 1). The markedly strong (P < 0.05) antioxidant response of TLC and TLE in comparison with ascorbic acid might be helpful in characterizing the significant sources of natural antioxidant reaction.

### Hydrogen peroxide radical scavenging activity

The scavenging effect of different fractions of *T*. *leptophylla* on hydrogen peroxide was concentration-dependent (25 - 500 μg/ml) as shown in Figure 1E. TLE displayed strong H_2_O_2_ scavenging activity (EC_50_ 68.0±2 μg/ml) whereas that of the standard, ascorbic acid exhibited 9.0±0.3 μg /ml. The scavenging activities of TLC and TLM were (EC_50_ 122±5 μg/ml and 130±5 μg/ml, respectively) (Table 1). EC_50_ values of the fractions in scavenging hydrogen peroxide were significantly different (P < 0.05) from the EC_50_ values obtained for ascorbic acid. The scavenging activity for hydrogen peroxide of various solvent extracts from *T*. *leptophylla* was in the order of TLE > TLC > TLM > TLH > TLB > TLA respectively.

### ABTS radical scavenging activity

All the fractions of *T*. *leptophylla* scavenged ABTS radical in a concentration-dependent way (25 - 250 μg/ml) (Figure 1F). Present results showed that the ABTS radical scavenging ability of samples can be ranked as TLB > TLA > TLE > TLH > TLC > TLM. TLB and TLA exhibited prominent ABTS radical scavenging activities.

### Reducing power activity

**Figure 2:**
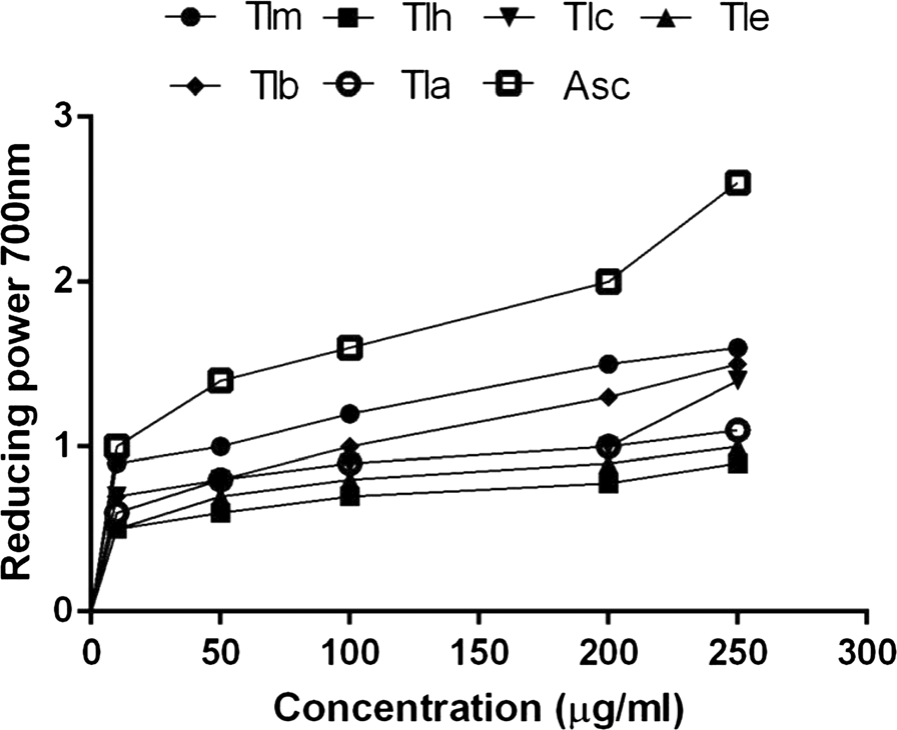
**Reducing power of different extracts from the methanol extract of *T. leptophylla *by different solvents at different concentrations.** Each value represents a mean ± SD (n = 3). Tlm., methanolic fraction, Tlh., *n*-hexane fraction, Tlc., chloroform fraction, Tle., ethyl acetate fraction, Tlb., *n*-butanol fraction, Tla., residual aqueous fraction, Asc., ascorbic acid.

Figure 2 shows the dose response curves for the reducing powers of all extracts (25 - 250 μg/ml) from *T*. *leptophylla*. It was found that the reducing power increased with concentration of each sample. The ranking order for reducing power was TLM > TLB > TLC > TLA > TLE > TLH. Significantly higher reducing power (1.47±0.14 at 250 μg/ml) was evident in TLM fraction.

### Extraction yield, total phenolic and flavonoid contents

The extraction yield of different fractions of *T*. *leptophylla* varied from 4.3±0.4 to 11.5±1.6% with a descending order of TLM > TLA > TLE > TLH > TLB > TLC (Table 2). So the extraction with methanol resulted in the highest amount of total extractable compounds whereas the extraction yield with chloroform was only small in comparison with that of the other solvents.

**Table 2:** **Total phenolics, flavonoid and extraction yield of methanol extract and soluble fractions of *T. leptophylla ***

**Plant extracts**	**Total phenolics (mg gallic acid equivalent/g)**	**Total flavonoid (mg rutin equivalent/g)**	**Extraction yield (%)**
Methanol extract	121.9±3.1^c^	59.6±1.5^c^	11.5±1.6^d^
*n*-Hexane fraction	54.9±2.8^a^	15.8±0.9^a^	5.4±1.3^b^
Chloroform fraction	78.0±1.1^b^	26.0±1.9^b^	4.3±0.4^a^
Ethyl acetate fraction	80.0±5.2^b^	60.9±2.2^c^	6.1±0.8^b^
*n*-Butanol fraction	80.9±2.9^b^	55.0±2.5^c^	4.8±1.1^a^
Residual aqueous fraction	49.9±4.1^a^	14.9±0.4^a^	8.2±0.9^c^

Each value in the table is represented as mean ± SD (n = 3). Values in the same column followed by a different letter (^a-d^) are significantly different (P < 0.05).

Total phenolic content was estimated by using Folin-Ciocalteu reagent. Total phenolic content of the different fractions of *T*. *leptophylla* was solvent dependent and expressed as milligrams of gallic acid equivalents (GAE) equivalent. Table 2 summarizes that total phenolic compounds in fractions varied widely, ranging from 49.9±4.1 and 121.9±3.1 mg/g expressed as gallic acid equivalents (GAE). TLM exhibited the highest total phenolic content. The content of flavonoid expressed as rutin equivalents, varied from 14.9±0.4 to 60.9±2.2 mg rutin equivalent/g extract (Table 2). The TLE showed the highest amount of flavonoid contents followed by TLM and TLB.

### Correlation of EC_50_ values of antioxidant activities with TPC and TFC

For total phenolic content a significant but marginal positive correlation (R^2^ > 0.7020, R^2^ > 0.7458, R^2^ > 0.6663 and R^2^ > 0.6777 respectively) was found between TPC and EC_50_ values for DPPH, hydroxyl, phosphomolybdate and ABTS respectively, while also another weak positive correlation was found between TFC and EC_50_ values for superoxide anion and hydroxyl radicals. However, a non significant correlation was found in case of hydrogen peroxide radical scavenging with both TPC and TFC (Table 3).

**Table 3:** **Correlations between the EC**_**50**_ **values of antioxidant activities and phenolic and flavonoids content of** ***T. leptophylla ***

**Assays**	**Correlation R**^**2**^	
	**Phenolics**	**Flavonoid**
EC_50_ of DPPH radical scavenging ability	0.7020*	0.192
EC_50_ of superoxide radical scavenging ability	0.1905	0.6613*
EC_50_ of phosphomolybdate antioxidant capacity	0.6663*	0.4306
EC_50_ of hydroxyl radical scavenging ability	0.7458*	0.7215*
EC_50_ of hydrogen peroxide radical scavenging ability	0.0194	0.0126
EC_50_ of ABTS radical scavenging ability	0.6777*	0.1285

Each value in the table is represented as mean ± SD (n = 3). * indicates significance at P < 0.05.

### *In vivo* antioxidant activity

*In vivo* lipid peroxidation study reveals that rats of CCl_4_ treated group showed significant increase (P < 0.05) in thiobarbituric acid reactive substances (TBARS) when compared with rats of normal control group. TLM and silymarin were able to significantly blunt (P < 0.05) this rise in TBARS level (Table 4). There was a marked decrease in the level of GSH in CCl_4_ treated group when compared with normal control group. The GSH level was significantly increased (P < 0.05) in TLM and silymarin treated groups (Table 4).

**Table 4:** Effects of TLM on hepatic protein, lipid peroxidation (TBARS) and glutathione (GSH)

**Group**	**Protein (μg/mg tissue)**	**TBARS (nM/mg protein)**	**GSH (μg/mg protein)**
1	1.697±0.024+	3.43 ± 0.53+	31.34 ± 1.10+
II	0.657±0.021*	6.39 ± 0.98*	15.99 ± 1.67*
III	1.563±0.092*+	4.30 ± 0.91*+	26.68 ± 1.16*+
IV	1.509±0.003+	4.69 ± 0.74*+	27.42 ± 1.21*+
V	1.702±0.012+	3.67 ± 0.42+	29.97 ± 1.12+

Group I: Controls (Olive oil+ DMSO); II: CCl_4_ (3 ml/kg bw); III: TLM (200 mg/kg bw) +CCl_4_ (3 ml/kg bw); IV: Silymarin (50 mg/kg bw) +CCl_4_ (3 ml/kg bw); V: TLM (200 mg/kg bw).

TLM; methanol extract of *T*. *leptophylla*.

Values are Mean ± SD (06 numbers).

*, Indicate significance from control group (P < 0.05).

+, Indicate significance from CCl_4_ group (P < 0.05).

## Discussion

Several techniques have been used to determine the antioxidant activity *in vitro* in order to allow rapid screening of substances since substances that have low antioxidant activity *in vitro*, will probably show little activity *in vivo*[8]. Free radicals are known to play a definite role in a wide variety of pathological manifestations. Antioxidants fight against free radicals and protect us from various diseases. They exert their action either by scavenging the reactive oxygen species or protecting the antioxidant defense mechanisms [21].

The electron donation ability of natural products can be measured by 2,2^′^-diphenyl-1- picrylhydrazyl radical (DPPH) purple-coloured solution bleaching [8]. The method is based on scavenging of DPPH through the addition of a radical species or antioxidant that decolourizes the DPPH solution. The degree of colour change is proportional to the concentration and potency of the antioxidants. A large decrease in the absorbance of the reaction mixture indicates significant free radical scavenging activity of the compound under test [36]. In the present study among all the fractions tested, *n*-butanol, chloroform and ethyl acetate showed significantly higher inhibition percentage and positively correlated with total phenolic content. Results of this study suggest that the plant extract contain phytochemical constituents that are capable of donating hydrogen to a free radical to scavenge the potential damage.

Superoxide radical is considered a major biological source of reactive oxygen species [34]. Although superoxide anion is a weak oxidant, it gives rise to generation of powerful and dangerous hydroxyl radicals as well as singlet oxygen, both of which contribute to oxidative stress [35]. The results of our study revealed that TLM, TLC and TLB had effective capacity of scavenging for superoxide radical and correlated with total flavonoid content thus suggesting its antioxidant potential.

The antioxidant capacity of the fractions was measured spectrophotometrically through phosphomolybdenum method, based on the reduction of Mo (VI) to Mo (V) by the test sample and the subsequent formation of green phosphate/Mo (V) compounds with a maximum absorption at 765 nm. The present study demonstrated that TLB exhibited the highest antioxidant capacity for phosphomolybdate reduction. Recent studies have shown that many flavonoid and related polyphenols contribute significantly to the phosphomolybdate scavenging activity of medicinal plants [37,38].

Hydroxyl radical is one of the potent reactive oxygen species in the biological system. It reacts with polyunsaturated fatty acid moieties of cell membrane phospholipids and causes damage to cell [5,39]. The hydroxyl radical is regarded as a detrimental species in pathophysiological processes and capable of damaging almost every molecule of biological system and contributes to carcinogenesis, mutagenesis and cytotoxicity [40]. Hydroxyl radicals were produced by the reaction of H_2_O_2_ and the ferrous that would react with 2-deoxyribose. The reaction was stopped by adding TBA reagent that would give a red colour if the malonaldehyde was formed as the result of the reaction between the radical and 2-deoxyribose. Hydroxyl radical scavenging capacity of an extract is directly proportional to its antioxidant activity which is depicted by the low intensity of red colour [41]. All fractions of *T*. *leptophylla* when added to the reaction mixture actively scavenged the hydroxyl radicals and prevented the degradation of 2-deoxyribose.

Hydrogen peroxide occurs naturally at low concentration levels in the air, water, human body, plants, microorganisms and food [41]. H_2_O_2_ is rapidly decomposed into oxygen and water and this may produce hydroxyl radicals (·OH) that can initiate lipid peroxidation and cause DNA damage [42]. Ethyl acetate fraction of *T*. *leptophylla* efficiently scavenged hydrogen peroxide which may be attributed to the presence of phenolic groups that could donate electrons to hydrogen peroxide, thereby neutralizing it into water.

ABTS radical scavenging assay involves a method that generates a blue/green ABTS^+^ chromophore via the reaction of ABTS and potassium persulfate. The ABTS radical cation is generated by the oxidation of ABTS with potassium persulfate, its reduction in the presence of hydrogen-donating antioxidants is measured spectrophotometrically at 745 nm. All the fractions possessed strong ABTS scavenging activity an observation that is supported by other researchers [43].

In reducing power assay, the yellow colour of the test solution changes to green depending on the reducing power of the test specimen. The presence of the reductants in the solution causes the reduction of the Fe^3+^/ferricyanide complex to the ferrous form. Therefore, Fe^2+^ can be monitored by absorbance measurement at 700 nm. Previous reports suggested that the reducing properties have been shown to exert antioxidant action by donating of a hydrogen atom to break the free radical chain [44]. Increasing absorbance at 700 nm indicates an increase in reducing ability. The antioxidants present in the fractions of *T*. *leptophylla* caused their reduction of Fe^3**+**^**/** ferricyanide complex to the ferrous form, and thus proved the reducing power.

Plant materials rich in phenolics are increasingly being used in the food industry because they retard oxidative degradation of lipids and improve the quality and nutritional value of food [45]. Phenolic compounds are considered secondary metabolites and these phytochemical compounds derived from phenylalanine and tyrosine occur ubiquitously in plants and are diversified [46]. The methanol extract exhibited the highest total phenolics content, whereas the contents obtained with residual aqueous fraction were much smaller that is in agreement with other reports [47]. Phenolic compounds of plants are also very important because their hydroxyl groups confer scavenging ability.

Phenolic compounds of plants fall into several categories; chief among these are the flavonoids which have potent antioxidant activities [8]. Flavonoids are naturally occurring in plants and are thought to have positive effects on human health. Studies on flavonoidic derivatives have shown a wide range of antibacterial, antiviral, anti inflammatory, anticancer, and anti-allergic activities [48,49]. Flavonoids have been shown to be highly effective scavengers of most oxidizing molecules, including singlet oxygen, and various free radicals [50] implicated in several diseases. So comparable with the findings in the literature for other extracts of plant products [42] our results suggested that phenolic acids and flavonoids may be the major contributors for the antioxidant activity as the EC_50_ values of radical scavenging activity of various soluble fractions of *T*. *leptophylla* and the contents of phenolics or flavonoids exhibited significant correlation. However, non significant correlation was found in case of hydrogen peroxide radical scavenging. It is known that different phenolic compounds have different responses in the Folin-Ciocalteu method. Similarly the molecular antioxidant response of phenolic compounds varies remarkably, depending on their chemical structure [51]. In addition, there may be some interference rising from other chemical components present in the extract, such as sugars or ascorbic acid [52].

Liver damage is very common since liver has to detoxicate a lot many toxic substances. There are several chemicals that have been known to induce hepatotoxicity by producing the reactive species which form covalent bonds with the lipids of the tissue [53,54]. Liver injury due to CCl_4_ in rats was first reported in 1936 [55] and has been widely and successfully used by many investigators [56,57]. Carbon tetrachloride is metabolized by cytochrome P-450 in endoplasmic reticulum and mitochondria with the formation of CCl_3_O^-^, a reactive oxidative free radical, which initiates lipid peroxidation [58,59].

A large reserve of reduced glutathione is present in hepatocytes for detoxification of free radicals. However, oxidative stress results in toxicity when the rate at which the ROS are generated exceeds the cell capacity for their removal [60,61]. Most hepatotoxic chemicals damage liver by inducing, directly or indirectly, lipid peroxidation. MDA is one of the end products in the lipid peroxidation process [62]. In our *in vivo* study elevation in levels of end products of lipid peroxidation in liver of rats treated with CCl_4_ were observed. The increase in MDA levels in liver suggests enhanced lipid peroxidation leading to tissue damage [63]. Treatment with TLM significantly reversed these changes. Hence it may be possible that the mechanism of hepatoprotection of extract is due to its antioxidant effect [63].

GSH is an intracellular reductant and protects cells against free radicals, peroxides and other toxic compounds. GSH is a naturally occurring substance that is abundant in many living creatures; GSH depletion increases the sensitivity of cells to various aggressions leading to tissue disorder and injury [64]. In the present study we demonstrated the effectiveness of the extract by using CCl_4_ induced rats and found that exogenous TLM supplementation elevated GSH levels in rats with CCl_4_ treatment and thus might provide a mean of recovering reduced GSH levels and to prevent tissue disorders and injuries. Therefore, it is valid to consider that TLM, because of its antioxidant property as the extract has confirmed the presence of saponins, might be capable of protecting the hepatic tissue from CCl_4_-induced injury and inflammatory changes. Saponins are natural products, which have been shown to possess antioxidant properties [65,66].

## Conclusion

The replacement of synthetic with natural antioxidants (because of implications for human health) may be advantageous. In the present study analysis of free radical scavenging activity and total phenolic and flavonoid content showed that mainly the TLB, TLC, TLE and TLM from the whole plant of *T*. *leptophylla* can be the potent source of natural antioxidants. The results of *in vivo* studies suggest that methanolic extract of *T*. *leptophylla* may be useful in defense against CCl_4_-induced liver damage possibly be due to its antioxidant properties.

## Competing interests

The authors declare that they have no competing interests.

## Authors’ contributions

NS made significant contribution to acquisition of data, analysis, drafting of the manuscript. MRK has made substantial contribution to conception and design, interpretation of data, drafting and revising the manuscript for intellectual content. MS participated in the design and collection of data and analysis. All authors read and approved the final manuscript.

## Pre-publication history

The pre-publication history for this paper can be accessed here:

http://www.biomedcentral.com/1472-6882/12/221/prepub
